# Medical and Philosophical News

**Published:** 1781-06

**Authors:** 


Hitherto but little ſatisfactory information has been derived from meteorological diaries. It is probable, however, that by comparing obſervations of this kind made in a great number of different places at the ſame time, ſome uſeful facts might be eſtabliſhed. It is with pleaſure, therefore, we inform our readers, that ſeveral ſocieties have lately been inſtituted for this purpoſe. One of theſe is at Carlſruhe, under the patronage of the Margrave of Baden-Dourlach. Profeſſor Brökman is appointed director of this new eſtabliſhment, and the neceſſary obſervations are to be made at ſixteen different places in the territories of Baden.—The Elector Palatine, it ſeems, has ſince adopted a ſimilar plan in his dominions; and at the Hague, a medico-meteorological ſociety has lately been eſtabliſhed with the ſame views.

